# PARTICIPATORY DESIGN OF A WEB-BASED COMMUNITY AND RESOURCE CONNECTION TOOL FOR RURAL DEMENTIA FAMILY CAREGIVERS

**DOI:** 10.1093/geroni/igad104.1885

**Published:** 2023-12-21

**Authors:** Nicole Werner

**Affiliations:** Indiana University School of Public Health-Bloomington, Bloomington, Indiana, United States

## Abstract

Rural family caregivers of people living with dementia have higher caregiver burden and experience lower social support than their urban counterparts. Technology has potential to address rural caregiver health disparities, but only if designed to their specific needs. Available technology interventions lack focus on rural caregivers and community influence. This represents a gap in rural caregivers’ ability to leverage technology for community supports to overcome their unique barriers. Thus, we are engaging rural caregivers and community resources in a participatory co-design process to design CareVirtue Family Connection, an online community and personalized resource connection tool specific to rural dementia caregivers. We conducted N=19 interviews with dementia caregivers self-identified as living in a rural area. We analyzed transcripts using team-based affinity diagramming to create meaningful clusters related to how rural caregivers get the help they need. We used consensus discussion to refine clusters and applied resulting categories to transcripts to identify confirming/disconfirming evidence. We identified seven dimensions influencing rural caregivers’ ability to get the help they need including community attributes, caregiving challenges, community connection, experience with community supports, caregiving goals, attitudes on caregiving and community, and information behavior. Results will guide subsequent co-design in which we will engage rural caregivers and related community organizations to co-design the CareVirtue Family Connection tool. Our participatory approach is aligned with the National Strategy to Support Family Caregivers’ goal to advance caregiver engagement, and CareVirtue Family Connection will support the Strategy’s vision to “expand access to programs, services, supports, and products to family caregivers.”

